# Surgical repair of acute Achilles tendon rupture with and without human amniotic membrane augmentation: a retrospective comparative study

**DOI:** 10.3389/fsurg.2025.1654648

**Published:** 2025-10-09

**Authors:** Ju Chun Chien, Meng Chen Kuo, Yi Jiun Chou, Yi Ping Wei

**Affiliations:** 1 Department of Radiation Oncology, Kaohsiung Veteran General Hospital, Kaohsiung, Taiwan; 2 Office of R&D with International Affairs, Tajen University, Pingtung City, Taiwan; 3 Department of Orthopedics, Kaohsiung Veterans General Hospital, Kaohsiung, Taiwan; 4 Institute of Biomedical Sciences, National Sun Yat-sen University, Kaohsiung, Taiwan; 5 Department of Occupational Therapy, Shu-Zen Junior College of Medicine and Management, Kaohsiung, Taiwan

**Keywords:** Achilles tendon rupture, amniotic membrane, biological augmentation, tendon healing, orthopedic surgery, regenerative medicine

## Abstract

**Objectives:**

Achilles tendon ruptures are common, particularly in active individuals; however, optimal healing remains challenging due to limited vascularity, adhesions, and re-rupture risk. Human amniotic membrane (hAM), rich in extracellular matrix and bioactive factors, shows regenerative potential, offering a promising adjunct for tendon repair.

**Methods:**

After institutional review board approval, patients aged 20–75 years who underwent end-to-end Achilles tendon repair for acute complete rupture between February 2019 and January 2025 were retrospectively reviewed. Patients were grouped by intraoperative use of hAM allografts. Baseline characteristics, rupture location, operative time, complications (tendon re-rupture, wound infection, dehiscence), American Orthopaedic Foot and Ankle Society (AOFAS) Ankle-Hindfoot score, and follow-up duration were analyzed using *t*-tests and Fisher's exact tests (*p* < 0.05).

**Results:**

In total, 45 repairs were performed in 44 patients. Of these, 13 patients (13 feet) received hAM augmentation, while 31 patients (32 feet) underwent primary repair without hAM. The overall mean follow-up time for the entire cohort was 38.16 ± 21.13 months. Complications occurred in 11 of the 45 feet, including 1 wound dehiscence, 3 re-rupture (2 of which were associated with wound infection), and 7 additional cases of wound infection. Operative time was significantly shorter in the hAM group (94.00 ± 30.12 vs. 116.72 ± 24.46 min, *p* = 0.011), with no significant differences in complication rates or AOFAS scores. Ruptures closer to the calcaneal insertion were associated with higher infection risk (2.86 ± 1.41 cm vs. 4.36 ± 1.14 cm, *p* = 0.002). Complications correlated with lower AOFAS scores (*p* < 0.0001), independent from hAM use.

**Conclusions:**

The hAM augmentation reduced operative time without affecting complication rates or functional outcomes. Further prospective studies are needed to confirm its clinical benefits.

Level of evidence: IV.

## 1 Introduction

The Achilles tendon, the strongest and thickest tendon in the human body, connects the gastrocnemius and soleus muscles to the calcaneus and plays a vital role in plantarflexion and lower limb function (1). Despite its strength, it is the most commonly ruptured tendon of the lower extremity, particularly in active individuals aged 30–50 (2). Most ruptures occur 2–6 cm proximal to the calcaneal insertion, an area with poor vascularity (3). Contributing factors include sudden eccentric loading, overuse in sports, systemic diseases (e.g., diabetes, renal dysfunction), corticosteroid or fluoroquinolone use, and age-related degenerative changes (4).

In complete tendon ruptures, particularly in patients with high functional demands, surgical repair is typically recommended (5). While conventional tenorrhaphy restores tendon continuity, challenges remain in promoting optimal healing, reducing adhesion formation, and minimizing re-rupture risk. These limitations have prompted interest in biological augmentation strategies to enhance surgical outcomes.

Human amniotic membrane (hAM) has emerged as a promising adjunct in tendon repair due to its unique biological properties (6). The amniotic membrane is of embryonic origin and is rich in extracellular matrix (ECM) proteins, collagen, and a range of growth factors such as transforming growth factor-beta 1 (TGF-β1), interleukin (IL) −1 receptor antagonist, platelet-derived growth factor (PDGF), and insulin-like growth factor (IGF) (6, 7). These factors promote cell migration, modulate inflammation, and support tissue regeneration (6). Structurally, hAM consists of an epithelial layer, basement membrane, and stromal matrix that together create an immunoprivileged, anti-inflammatory scaffold capable of minimizing fibrosis while facilitating repair (8).

Preclinical studies have demonstrated the regenerative potential of amniotic-derived cells and matrices in tendon healing (6, 9). Application of amniotic epithelial cells in animal models of tendon injury has been shown to improve histological organization and biomechanical strength (10). Similarly, injections of amniotic fluid-derived cells have reduced adhesions and enhanced tensile properties in repaired tendons (11). Although the exact mechanism of Achilles tendon rupture remains unclear, ruptures typically occur in tendons with pre-existing but asymptomatic abnormalities, potentially involving dysvascularity, which may render them responsive to adjunctive biologic therapies (12, 13).

In clinical practice, dehydrated human amnion/chorion membrane allografts have been used successfully in chronic tendinopathy and other orthopedic conditions, resulting in improved pain control and function without adverse effects (14, 15).

Given these properties, hAM presents a biologically active scaffold that may enhance surgical repair in Achilles tendon ruptures of varying chronicity. This study aims to evaluate and compare the outcomes of Achilles tendon repair with and without hAM augmentation in a retrospective cohort. We hypothesize that hAM incorporation will promote more organized tendon healing, and improve functional outcomes.

## 2 Methods

After obtaining approval from the research ethics board (KSVGH25-CT4-14), the author conducted a retrospective comparative study by reviewing the institutional database for patients who underwent Achilles tendon repair between February 2019 and January 2025 due to acute complete rupture. This study was conducted at a level-one medical center.

All patients aged 20–75 years with a closed Achilles tendon rupture who presented to our institution were prospectively screened for inclusion. The diagnosis was established based on medical history (typically associated with high-stress physical activities, prolonged exercise, or traumatic events) and clinical examination, including the presence of a palpable gap and a positive Thompson test. In cases where physical examination was inconclusive, additional imaging, primarily ultrasonography, was performed to confirm the presence and extent of the rupture. Intraoperative findings were used to verify the diagnosis, and only patients with a confirmed complete rupture were enrolled.

Only patients who underwent end-to-end Achilles tendon repair were included. Patients who underwent flexor hallucis longus (FHL) transfer, combined V–Y advancement procedures, or tendon reattachment using suture anchors were excluded.

Additional exclusion criteria included distal Achilles tendon tears (insertional ruptures) or avulsions, ruptures older than 4 weeks, a history of ipsilateral Achilles tendinopathy or prior ipsilateral Achilles tendon-related surgery, previous lower extremity injuries impairing limb function, neuromuscular disorders, peripheral vascular disease, active skin infections or wounds, and inability to comply with postoperative rehabilitation or follow-up assessments.

The decision to use hAM augmentation was not randomized. Instead, a shared decision-making approach was adopted. Prior to surgery, the operating surgeon informed patients about the indications, potential benefits, and additional costs associated with hAM allograft augmentation. Based on this discussion, patients made the final decision regarding whether to receive hAM augmentation, and their preference was respected. As a result, the control group was not randomized but consisted of patients who elected to undergo primary repair without hAM augmentation.

### 2.1 Data collection and outcome assessment

Medical records were systematically reviewed to collect baseline patient data (including height, weight, and medical history), preoperative clinical findings, operative records (including the location of tendon rupture and operative time), and to identify postoperative complications, such as infection, tendon re-rupture, and wound dehiscence. Functional outcomes were assessed using the American Orthopaedic Foot and Ankle Society (AOFAS) Ankle-Hindfoot score (16). Follow-up assessments were conducted either during outpatient clinic visits or via telephone interviews. The follow-up period was defined as the interval from surgery to the date of the latest clinical evaluation or telephone interview.

The same postoperative protocol was applied to all patients. An initial 2-week period of non-weight bearing immobilization in a cast with the ankle positioned in resting equinus (15–20 degrees of plantar flexion) was followed by a 4-week phase of progressive weight bearing. Subsequently, between postoperative weeks 7 and 10, patients transitioned to full weight-bearing ambulation in a walking boot with the ankle positioned at neutral dorsiflexion (0 degree). No aggressive stretching of the Achilles tendon or the gastrocnemius-soleus complex was permitted before 12 weeks postoperatively.

Data analysis was performed according to variable type and distribution using descriptive statistical methods. All statistical analyses were conducted using SPSS Statistics version 20 (IBM Corp., Armonk, NY, USA). Continuous variables were presented as mean ± standard deviation (SD) and compared using two-sample t-tests. Categorical variables were compared using Fisher's exact tests. A *P* value of <0.05 was considered statistically significant.

The study first compared patients who received hAM augmentation and those who did not, evaluating baseline characteristics, time from injury to surgery, operative time, follow-up duration, and clinical outcomes (including complication rates and postoperative AOFAS scores). Subsequently, comparisons were performed between patients who developed postoperative complications (tendon re-rupture or wound infection) and those who did not, to assess potential factors associated with complications and their impact on functional outcomes.

### 2.2 Surgical techniques

Patients were surgically treated by one of seven experienced orthopedic surgeons, all of whom had received standardized surgical training at the same medical center. Prophylactic antibiotics (cefazolin; clindamycin was administered in cases of cefazolin allergy) were given preoperatively. Surgery was performed with the patient in the prone position under tourniquet control. A longitudinal posteromedial skin incision was made over the rupture site, and the paratenon was carefully identified and incised.

The tendon was repaired end-to-end using core sutures with a hybrid technique incorporating elements of the modified Kessler, Krackow, and Bunnell methods, utilizing NO. 2-0 Ti-Cron (Covidien, Mansfield, MA) sutures. Initially, sutures from all three techniques were carefully placed away from the rupture site and anchored into healthy tendon tissue (17). The Krackow sutures were then sequentially tightened to approximate the tendon ends and achieve optimal stability, followed by tightening of the remaining sutures in sequence.

The ankle was positioned in plantar flexion to approximate the tendon ends while avoiding overtension, with a maximum plantar flexion angle of 20 degrees to accommodate the brace. Reinforcement of the core repair was performed with a running circumferential epitendinous suture using #1 Vicryl (Ethicon, Somerville, NJ). Whenever possible, the paratenon was carefully approximated and repaired using 2-0 Polysorb sutures (Covidien, Mansfield, MA, USA) to provide coverage for the repaired tendon and facilitate the biological healing process, and skin closure was achieved using interrupted 3-0 nylon sutures. Postoperatively, the ankle was immobilized in a cast maintaining 15–20° of plantar flexion. On the first postoperative day, a window was created over the incision site using a cast saw to facilitate wound inspection and dressing changes.

### 2.3 Biological augmentation was performed intraoperatively by applying an allogeneic hAM graft to enhance the surgical repair

**Figure 1:**
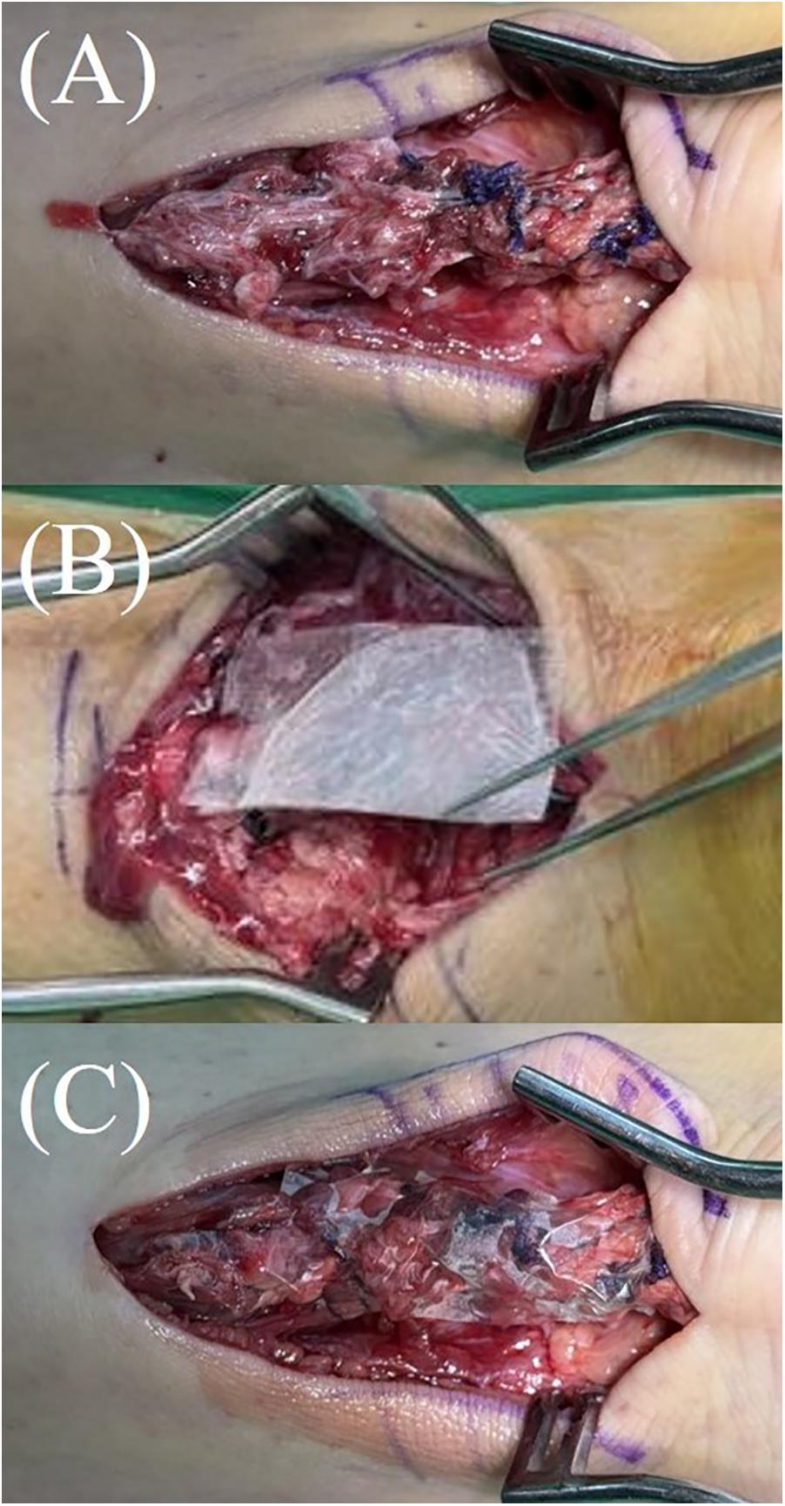
Application of hAM allograft during Achilles tendon repair. **(A)** The Achilles tendon after completion of end-to-end repair. **(B,C)** A 2 cm × 4 cm double-layer hAM allograft (AmnioGen, HCT Regenerative, Taiwan) applied and wrapped around the repaired tendon.

Once the Achilles tendon was reduced and repaired using core sutures, the Thompson test was performed to assess functional integrity. Following confirmation of adequate repair and thorough irrigation of the wound, a 2 cm × 4 cm double-layer hAM allograft (AmnioGen, HCT Regenerative, Taiwan) was applied to the repair site and wrapped around the repaired tendon, with the stromal layer facing the tendon and the epithelial layer oriented outward (Figure 1).

The double-layer hAM allograft (AmnioGen, HCT Regenerative, Taiwan) used in this study is a pre-fabricated, sheet-type commercial product. It is supplied in sterile packaging as ready-to-use grafts. Immediately prior to implantation, the dehydrated human amniotic membrane allograft was removed from its sterile packaging and directly applied to the repair site without any additional laboratory processing. As illustrated in Figures 1B,C, the material was already in sheet form and could be wrapped around the repaired tendon after thorough wound irrigation and confirmation of adequate repair.

The allograft was utilized to provide both biological support and mechanical protection to the repaired tendon, especially in cases where the paratenon was severely compromised and could not be sufficiently reconstructed. The incision was then closed, and the patient was placed in a posterior short-leg splint with the ankle maintained at approximately 20 degrees of plantarflexion.

### 2.4 Human amniotic membrane collection and processing

Commercially available hAM allografts were obtained from HCT Regenerative, a Taiwan Food and Drug Administration (TFDA)–licensed and American Association of Tissue Banks (AATB)–accredited tissue bank (18). Placental tissue was recovered in Taiwan through informed maternal consent and processed under standardized protocols for clinical use. All donors underwent comprehensive risk assessment and serological/nucleic acid testing (Anti–HIV-1/2, HIV-1 NAT, HBsAg, HBV NAT, Anti-HBc, Anti-HBs, Anti-HCV, HCV NAT, RPR, TPPA) in compliance with regulatory requirements (18). The final products were prepared in an ISO class 10,000 cleanroom and released as sterile, ready-to-use grafts (18). Microbiological testing was conducted before processing and after packaging. All steps complied with TFDA licensure and AATB-accredited tissue banking standards, ensuring clinical safety and reproducibility.

## 3 Results

Between February 2019 and January 2025, 45 feet in 44 patients were included. In our cohort of patients with acute Achilles tendon rupture (defined as rupture ≤ 4 weeks), there were no documented cases of corticosteroid use, fluoroquinolone exposure, or patients undergoing dialysis. A total of 15 patients were excluded: 1 patient due to a history of peripheral arterial occlusive disease, 2 patients with partial Achilles tendon tears, and twelve patients underwent surgery more than 4 weeks after injury, among whom three required tendon reattachment procedures.

Mean follow-up was 38.16 ± 21.13 months (range, 6–72 months). The average interval between injury and surgery was 3.89 (range, 0–14) days.

During a minimum follow-up period of 6 months, complications occurred in 11 of the 45 feet, including 1 case of wound dehiscence, 3 cases of tendon re-rupture (2 of which were associated with wound infection), and 7 additional cases of wound infection.

One patient in the non-hAM group developed postoperative wound dehiscence, which healed completely after one month of outpatient follow-up and regular wound dressing changes.

Postoperative complete tendon re-rupture occurred in 3 of 45 feet (6.67%): one in the hAM group (1/13, 7.69%) and two in the non-hAM group (2/32, 6.25%).

One re-rupture was diagnosed based on clinical examination and magnetic resonance imaging (MRI) image findings, while the other two were identified intraoperatively during debridement procedures performed for concomitant infections. All three patients underwent additional surgical interventions, including debridement combined with direct tendon repair, or FHL transfer combined repair. Post-revision clinical examinations confirmed tendon healing in all three patients.

Postoperative wound infections occurred in nine feet (9/45, 20.0%), including one case in the hAM group (1/13, 7.69%) and eight cases in the non-hAM group (8/32, 25.0%). The onset of infection ranged from 1 week to 5 months postoperatively. The one infection case in the hAM group resolved with oral antibiotic treatment alone, whereas among the non-hAM group, one patient also recovered after oral antibiotics and the remaining seven required readmissions for surgical debridement; three of these underwent multiple procedures.

### 3.1 Comparison between hAM and non-hAM groups

Baseline characteristics and postoperative outcomes between patients who received hAM allograft augmentation (13 patients, 13 feet) and those who underwent primary tendon repair without hAM (31 patients, 32 feet) are summarized in Table 1. No significant differences were observed between the two groups regarding age, gender, height, weight, smoking status, diabetes mellitus, tendon rupture location, or time interval between injury and surgery. There was a significant difference in limb laterality (*p* = 0.047), with a higher proportion of left-sided ruptures in the hAM group. Operative time was significantly shorter in the hAM group (hAM: 94.00 ± 30.12 min vs. non-hAM: 116.72 ± 24.46 min, *p* = 0.011). The complication rates were not statistically different between groups, including postoperative tendon re-rupture (hAM: 7.69% vs. non-hAM: 6.25%, *p* = 1.000), wound infection (hAM: 7.69% vs. non-hAM: 25.00%, *p* = 0.249), and wound dehiscence (hAM: 0% vs. non-hAM: 3.13%, *p* = 1.000). Postoperative AOFAS scores were comparable (hAM: 80.15 ± 10.86 vs. non-hAM: 78.56 ± 7.43, *p* = 0.573). The follow-up period was significantly longer in the non-hAM group (hAM: 21.08 ± 12.59 months vs. non-hAM: 45.09 ± 20.00 months, *p* < 0.0001).

**Table 1:** Baseline characteristics of two groups: patients treated with amniotic membrane allograft vs. those without hAM allograft.

Type of surgery	Patients who received a hAM allograft	Patients who underwent primary tendon repair without the use of a hAM allograft	*p* value
Case number	13 (13 feet)	31 (32 feet)	
Age^a^	46.85 ± 15.43	44.81 ± 13.10	0.656^c^
Gender	2 Female; 11 Male	10 Female; 22 Male	0.460^d^
Left/Right limb	10l; 3R	13l; 19R	0.047^d^
Body height (in centimeters)^a^	171.32 ± 7.83	169.80 ± 9.91	0.625^c^
Body weight (kilograms)^a^	77.06 ± 23.17	75.82 ± 14.12	0.826^c^
Smoking habits (%)	2 (2/13 = 15.38%)	8 (8/32 = 25.00%)	0.698^d^
Presence of diabetes mellitus (%)	1 (1/13 = 7.70%)	0	0.289^d^
Location of tendon rupture (distance from calcaneal insertion site, in centimeters)^a^^,^^b^	4.71 ± 1.15	3.81 ± 1.34	0.063^c^
Time interval between injury and surgery (days)^a^	5.46 ± 5.10	3.25 ± 2.66	0.062^c^
Operative time^a^	94.00 ± 30.12	116.72 ± 24.46	0.011^c^
Postoperative tendon retear (%)	1 (1/13 = 7.69%)	2 (2/32 = 6.25%)	1.000^d^
Postoperative wound infection (%)	1 (1/13 = 7.69%)	8 (8/32 = 25.00%)	0.249^d^
Postoperative wound dehiscence (%)	0	1 (1/32 = 3.13%)	1.000^d^
AOFAS^a^	80.15 ± 10.86	78.56 ± 7.43	0.573^c^
Follow-up period (months)^a^	21.08 ± 12.59	45.09 ± 20.00	<0.0001^c^

AM, amniotic membrane; L, left; R, right; AOFAS, American orthopedic foot and ankle society.

a mean ± SD.

b The rupture location could not be identified in four patients because of incomplete medical documentation.

c Two-sample *t*-tests.

d Fisher's exact test.

### 3.2 Comparison between patients with and without complications

Comparisons between patients who developed postoperative tendon re-rupture (*n* = 3) and those who did not (*n* = 42) are shown in Table 2. No significant differences were observed regarding age, gender, limb side, height, weight, smoking status, diabetes mellitus, hAM usage, rupture location, time to operation, or operative time.

**Table 2:** Comparison of clinical characteristics between patients with and without postoperative tendon retear and infection.

Comparison of clinical characteristics between patients with and without postoperative tendon retear			
Variables	Patients who developed tendon retear	Patients without developed postoperative tendon retear	*p* value
Case number	3	42	
Age^a^	56.00 ± 14.73	44.64 ± 13.45	0.167^c^
Gender	1 female; 2 male	11 female; 31 male	1.000^d^
Left/Right limb	1L; 2R	22L; 20R	0.608^d^
Body height (in centimeters)^a^	167.33 ± 16.65	170.45 ± 8.86	0.581^c^
Body weight (kilograms)^a^	78.87 ± 10.28	75.98 ± 17.39	0.780^c^
Smoking habits (%)	1 (1/3 = 33.33%)	9 (9/42 = 21.43%)	0.539^d^
Presence of diabetes mellitus (%)	1 (1/3 = 33.33%)	0	0.067^d^
Case number of receiving an amniotic membrane allograft intraoperatively (%)	1 (1/3 = 33.33%)	12 (12/42 = 28.57%)	1.000^d^
Location of tendon rupture (distance from calcaneal insertion site, in centimeters)^a,b^	3.00 ± 2.60	4.11 ± 1.22	0.171^c^
Time interval between injury and surgery (days)^a^	2.67 ± 1.53	3.98 ± 3.72	0.551^c^
Operative time^a^	113.33 ± 37.53	109.93 ± 27.65	0.841^c^
AOFAS^a^	56.33 ± 10.97	80.64 ± 5.53	<0.0001^c^
Follow-up period (months)^a^	29.67 ± 25.15	38.76 ± 21.04	0.478^c^
**Comparison of clinical characteristics between patients with and without postoperative tendon infection**			
**Variables**	**Patients with developed postoperative tendon infection**	**Patients without developed postoperative tendon infection**	***p* value**
Case number	9	36	
Age^a^	51.67 ± 15.25	43.83 ± 13.00	0.125^c^
Gender	4 female; 5 male	8 female; 28 male	0.219^d^
Left/Right limb	2L; 7R	21L; 15R	0.071^d^
Body height (in centimeters)^a^	167.72 ± 7.87	170.87 ± 9.61	0.369^c^
Body weight (kilograms)^a^	84.80 ± 8.56	74.02 ± 17.90	0.088^c^
Smoking habits (%)	1 (1/9 = 11.11%)	9 (9/36 = 25.00%)	0.659^d^
Presence of diabetes mellitus (%)	0	1 (1/36 = 2.78%)	1.000^d^
Case number of receiving an amniotic membrane allograft intraoperatively (%)	1 (1/9 = 11.11%)	12 (12/36 = 33.33%)	0.249^d^
Location of tendon rupture (distance from calcaneal insertion site, in centimeters)^a,b^	2.86 ± 1.41	4.36 ± 1.14	0.002^c^
Time interval between injury and surgery (days)^a^	4.44 ± 2.01	3.75 ± 3.93	0.612^c^
Operative time^a^	123.56 ± 27.13	106.81 ± 27.41	0.108^c^
AOFAS^a^	70.33 ± 9.63	81.19 ± 6.67	<0.0001^c^
Follow-up period (months)^a^	40.78 ± 21.88	37.50 ± 21.20	0.682^c^

AOFAS, American orthopedic foot and ankle society.

a Mean ± SD.

b The rupture location could not be identified in four patients because of incomplete medical documentation.

c Two-sample *t*-tests.

d Fisher's exact test.

Comparisons between patients who developed postoperative tendon infection (*n* = 9) and those who did not (*n* = 36) are also presented in Table 2. Patients who developed infection had significantly more proximal rupture sites (closer to the calcaneal insertion) compared to those without infection (2.86 ± 1.41 cm vs. 4.36 ± 1.14 cm, *p* = 0.002). There were no significant differences in age, gender, height, weight, smoking status, diabetes mellitus, hAM usage, time to surgery, or operative time. Furthermore, patients who developed complications demonstrated significantly poorer functional outcomes. The mean AOFAS score was significantly lower in patients with tendon re-rupture (56.33 ± 10.97 vs. 80.64 ± 5.53, *p* < 0.0001) and tendon infection (70.33 ± 9.63 vs. 81.19 ± 6.67, *p* < 0.0001).

## 4 Discussion

The findings of this study demonstrated that: (1) the use of hAM allografts did not significantly affect overall complication rates or functional outcomes compared to primary repair without hAM, although a trend toward lower infection rates was observed in the hAM group; (2) the application of hAM was associated with a significantly shorter operative time, suggesting potential intraoperative advantages; and (3) rupture sites located more proximally, closer to the calcaneal insertion, were significantly associated with a higher risk of postoperative tendon infection. Furthermore, the presence of complications, including tendon re-ruptures and infection, was correlated with significantly poorer functional outcomes based on AOFAS scores.

Various orthobiologic materials have been investigated to augment Achilles tendon repair, including cellular therapies such as embryonic stem cells (ESCs), induced pluripotent stem cells (iPSCs), mesenchymal stromal/stem cells (MSCs), and tendon stem cells (TSCs), as well as acellular options such as platelet-rich plasma (PRP), hyaluronic acid (HA), bone marrow aspirate concentrate (BMAC), AM-derived products, and extracellular vesicles (EVs) (19, 20). These biologics exert their therapeutic effects through delivery of growth factors, cytokines, and stem cells that modulate inflammation, stimulate tenocyte proliferation, and promote extracellular matrix remodeling to enhance tendon regeneration (19, 20). While preclinical studies have demonstrated encouraging regenerative outcomes, current clinical evidence remains limited and heterogeneous, highlighting the need for standardized treatment protocols and high-quality prospective trials (6, 9). Table 3 summarizes the currently published orthobiologic augmentation strategies for Achilles tendon repair, with one representative recent study selected for each category to avoid redundancy (20–24).

**Table 3:** Current orthobiologic augmentation strategies for Achilles tendon repair.

Study (year)	Othobiologic therapies	Instructions for use	Mechanisms
Cem Zeki Esenyel et al. (21)	Hyalonect (a knitted mesh composed of HYAFF, a benzyl ester of hyaluronic acid)	Turndown gastrocnemius fascial flap and tendon were wrapped with a surgical mesh.	HA has been shown to have broad antiinflammatory, chondroprotective, analgesic, and proteoglycan synthetic effects.
Benjamin E. Stein et al. (22)	BMAC	Bone marrow aspirate was concentrated to yield a volume of 6–9 ml of BMAC. Following wound closure, the BMAC was injected directly into the repair site at various depths.	BMAC promotes tendon healing by releasing cytokines and growth factors that modulate inflammation, reduce fibrosis, and recruit tenocytes and mesenchymal stem cells. It contains hematopoietic and osteogenic growth factors such as VEGF, PDGF, and TGF-β that support tissue regeneration.
Thøger P. Krogh et al. (23)	PRP	PRP were injected using an antiseptic peppering technique by making 3–4 skin portals and about 7 tendon perforations evenly distributed in the thickest part of the tendon.	Concentrated platelets release growth factors and cytokines
Domenico Albano et al. (24)	PRP or SVF	A volume of 4 ml of either PRP or SVF was injected into Achilles tendon to ensure intratendinous delivery. PRP or SVF were injected in the most thickened area of the tendon, taking care to cover the whole area of degeneration.	Adipose-derived mesenchymal stem cellss are multipotent stem cells able to secrete growth factors that may accelerate healing as well as those released by PRP.
Varun Gopinatth et al. (20)	EVs	Studies on human Achilles tendons have not been performed.	The contents of exosomes facilitate cell-to-cell communication and paracrine signaling as well as mediate various metabolic pathways involved in cell proliferation, inflammation, angiogenesis, and chemotaxis.

HA, hyaluronic acid; BMAC, bone marrow aspirate concentrate; VEGF, vascular endothelial growth factor; PDGF, platelet-derived growth factor; TGF-β1, transforming growth factor-beta 1; PRP, platelet-rich plasma; SVF, adipose-derived stromal vascular fraction; EVs, extracellular vesicles.

**Figure 2:**
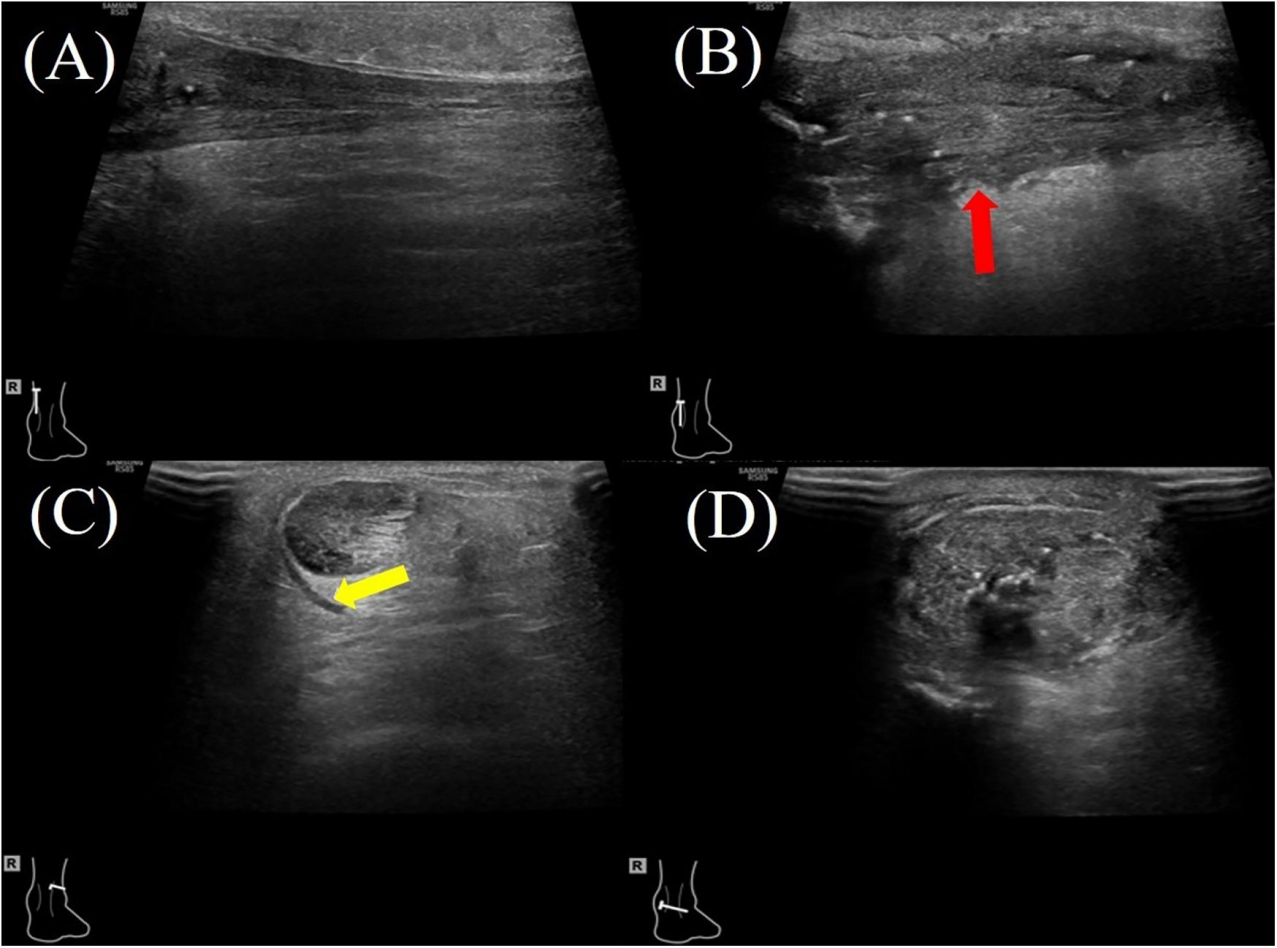
A 47-year-old female patient with right Achilles tendon rupture located 3.3 cm proximal to the calcaneal insertion underwent surgical repair with hAM augmentation. At the 2-month postoperative follow-up, ultrasound evaluation showed: **(A)** the proximal healthy tendon on the longitudinal plane showing a homogeneous echotexture. **(B)** the repair site (red arrow) on the longitudinal plane demonstrating mild heterogeneity in echotexture and variable tendon thickness; **(C)** the proximal healthy tendon on the transverse plane with a small amount of surrounding inflammatory effusion (yellow arrow). **(D)** the repaired tendon on the transverse plane, demonstrating increased cross-sectional area.

**Figure 3:**
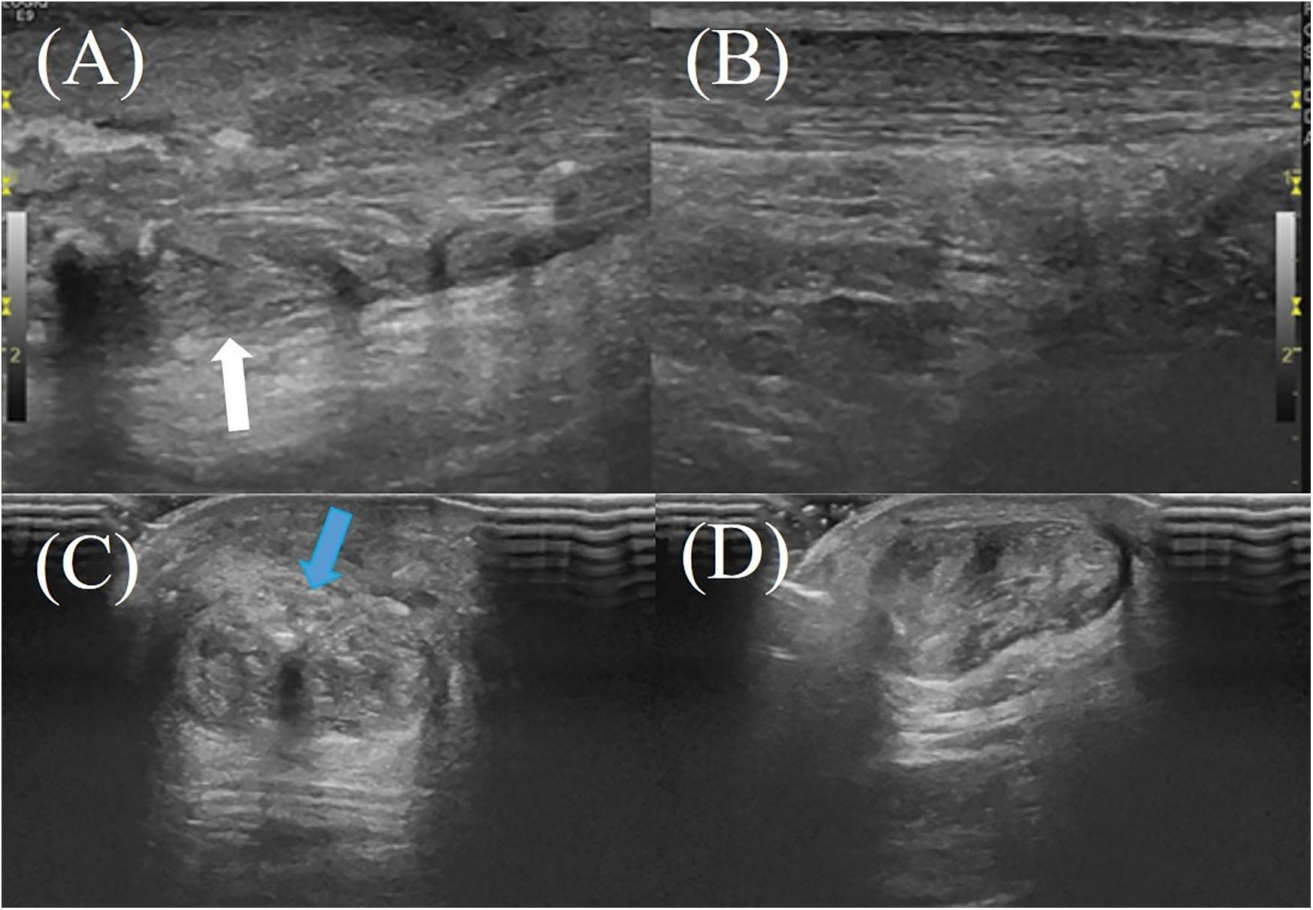
A 61-year-old male patient with right Achilles tendon rupture located 3.0 cm proximal to the calcaneal insertion underwent surgical repair without amniotic membrane augmentation. At the 2-month postoperative follow-up, ultrasound evaluation revealed: **(A)** the repaired tendon on the right side (white arrow) demonstrating heterogeneous echotexture on the longitudinal plane; **(B)** the healthy contralateral (left) Achilles tendon on the longitudinal plane; **(C,D)** the repaired right Achilles tendon on the transverse plane, showing irregular hyperechoic signals near the superficial tendon surface, indicating tendon fibrosis or degenerative changes (blue arrow).

The hAM, composed of multiple collagen types (I, III, V, VI), amniotic epithelial cells, and MSCs, secretes a wide range of bioactive cytokines and growth factors (e.g., TGF-β1, PDGF, IGF, IL-1, IL-10), which contribute to its anti-inflammatory, anti-fibrotic, and regenerative properties (6, 8, 20). In animal models, amniotic membrane application to Achilles tendon injuries has been shown to reduce inflammatory cell infiltration, improve type I and III collagen organization, and significantly enhance biomechanical properties such as tensile strength, stiffness, and cross-sectional area (25, 26) (Figures 2, 3). In contrast, the hAM allograft used in the present study was an acellular, non-vital material, which differs in biological composition from the materials evaluated in previous studies and may therefore not be directly comparable (25, 26). Further studies are needed to determine whether these differences influence clinical outcomes.

Preliminary human studies using amniotic membrane particulate or dehydrated allograft injections have demonstrated substantial pain reduction and functional improvement in Achilles tendinopathy patients (15, 27). However, larger, high-quality clinical trials are still required to further validate these findings. Table 4 summarizes the current literature regarding the clinical application of AM-derived products in Achilles tendon disorders and ruptures (15, 27–29).

**Table 4:** Application of AM-derived products in Achilles tendon disorders and ruptures.

Study(year)	AM-derived products	Patient population	Results
Bruce Werber (28)	PalinGen SportFLOW (Amnio Technology, llc. Phoenix, AZ)	Chronic plantar fasciosis and Achilles tendinosis	In patients with chronic plantar fasciosis and Achilles tendinosis unresponsive to standard therapies, treatment with granulized amniotic membrane and amniotic fluid significantly reduced pain from severe to mild within 12 weeks.
Jay E. Spector et al. (15)	mdHACM allograft	Achilles tendinopathy	mdHACM injection reduced or eliminated pain in all 32 patients with follow-up data
Michael J. Chin et al. (27)	AMUC (CLARIX FLO; Amniox; Miami, FL)	Achilles tendinopathy with or without a partial tear	AMUC injection significantly reduced pain and eliminated narcotic use in some patients within 3 months without treatment-related complications.
Mario Giacobazzi et al. (29)	Arthrex Amnion Matrix skin substitute (3 cm × 8 cm)	A 20-year-old athlete with prior contralateral Achilles tear presented with a full-thickness Achilles rupture.	By three months, the patient regained full ankle range of motion and resumed light activities without pain; by six months, he progressed well in physical therapy, regained motor control and balance, and was cleared for low-impact sports.

AM, amniotic membrane; mdHACM, micronized dehydrated human amnion/chorion membrane.

In our cohort study, we included patients who underwent Achilles tendon repair surgery at the same institution, and divided them into two groups based on the intraoperative use of hAM allografts. As shown in Table 1, the proportion of left-sided injuries was significantly higher in the hAM group, which may reflect bias due to the limited sample size. Additionally, the operative time was significantly shorter in the hAM group, with a mean reduction of 22.72 min. One possible explanation is that, in acute Achilles tendon injuries, the paratenon is often extensively damaged (30, 31). The hAM may serve as a substitute for the paratenon by covering the repaired tendon and partially restoring its protective function (32, 33). Patients in the hAM group also underwent paratenon repair; however, excessive suturing of severely damaged and fragile paratenon tissue was not required. In contrast, for patients without hAM augmentation, meticulous paratenon reconstruction is often required, which can be time-consuming and technically challenging, and in some cases, complete closure may not be achievable, leaving residual gaps.

The application technique was straightforward, involving simple wrapping of the graft around the repair site. The use of hAM allograft did not appear to substantially prolong operative time. However, this study was not designed to assess a potential learning curve effect. Because multiple surgeons were involved and the number of augmented cases was limited, it was not possible to determine whether operative efficiency was influenced by surgeon experience with hAM. Future studies may help clarify whether a learning curve exists when incorporating hAM augmentation.

Furthermore, the follow-up period was significantly shorter in the hAM group, as amniotic membrane products have only been introduced into clinical practice more recently, resulting in a shorter available observation period for these patients. However, the mean follow-up duration in this group still reached 21.08 months. Although the minimum follow-up duration was as short as 6 months in one patient, previous studies suggest this period is sufficient to evaluate postoperative complications and functional outcomes. One study reported that running and jogging at low mileage and intensity can begin as early as 13 weeks postoperatively, while a systematic review indicated that the average time to return to play was approximately 6 months (34, 35). Therefore, the follow-up period used in this study should be adequate to assess complications as well as AOFAS hindfoot scores, which incorporate pain, function (including walking ability), and alignment (16). Moreover, as shown in Table 2, there was no significant difference in follow-up duration between patients who developed complications (re-rupture or infection) and those who did not. Based on the findings summarized in Table 1, a trend toward lower infection rates was observed in the hAM group. This finding may be partially explained by the antimicrobial properties of AM, which contain various antimicrobial components such as bactricidin, β-lysin, lysozyme, transferrin, and 7-S immunoglobulins (36–38). In addition, the hAM allograft acts as a physical barrier that closely adheres to the tendon, potentially preventing microbial invasion (39). These combined biochemical and structural features may contribute to reducing the risk of postoperative infections following Achilles tendon repair.

As demonstrated in Table 2 of our cohort study, patients who developed postoperative infections had rupture sites located closer to the calcaneal insertion compared to those without infection. This association may be explained by two factors: (a) As the rupture site becomes more distal, the posteromedial skin incision tends to shift toward the midline due to limited skin mobility in the distal region. Although the ideal posteromedial incision should be made approximately 1 cm medial to the Achilles tendon, the reduced tissue mobility distally often necessitates an incision closer to the tendon (40). A cadaveric study using whole-body arterial perfusion and angiography demonstrated a longitudinal hypovascular area consistently present along the posterior midline overlying the Achilles tendon, whereas the medial and lateral regions adjacent to the tendon exhibit denser vascularity (41). Therefore, more distal rupture sites may require incisions closer to this hypovascular zone, potentially increasing the risk of wound complications and infection. In future surgical practice, the adjunctive use of hAM during wound closure may represent a potential strategy to enhance tissue mobility, given its antifibrotic properties. In addition, biologic materials such as amniotic/chorion membranes or umbilical cord grafts, which demonstrate stronger structural integrity and superior biomechanical characteristics, may offer further advantages in minimizing fibrosis and facilitating soft-tissue handling. However, these potential benefits require validation in future prospective studies (42). (b) Distal rupture locations may result in shorter remaining tendon stumps, necessitating more advanced surgical techniques and longer operative times to achieve stable fixation, both of which could contribute to a higher risk of infection.

Beyond its biological properties, the clinical success of hAM depends largely on its handling characteristics and technical feasibility during surgery. As described by Odet et al., hAM exhibits specific challenges such as folding upon detachment from its support and difficulty in maintaining correct orientation, which may require two surgeons for optimal application (43). Parameters such as ease of detachment, adhesion to bone, strength, suturability, and the possibility of burying the membrane between bone and mucosa are critical for reproducibility and user confidence. These practical considerations underscore that hAM is not only a biologically active graft but also a material that requires standardized training for appropriate intraoperative manipulation. Including these aspects may facilitate wider adoption and guide future users in tailoring hAM application strategies across surgical fields, from oral surgery to orthopedics.

In addition to its clinical use as a biologic scaffold, hAM has been increasingly investigated for its regenerative and multimodal properties. Consensus guidelines summarized by Pozzobon et al. highlight a range of functional assays designed to validate the activity of perinatal derivatives, including hAM (44). These assays encompass *in vivo* models of skeletal muscle regeneration, where decellularized hAM scaffolds were shown to enhance myofiber regeneration, restore tensile strength, and improve electrophysiological contractility. Beyond structural support, hAM and related derivatives have demonstrated paracrine effects such as immunomodulation, antifibrotic action, and pro-angiogenic signaling, which can be assessed by macrophage polarization studies, collagen deposition analysis, and functional readouts like grip or gait testing. Although no laboratory-based functional assays were performed in this study, the postoperative American Orthopaedic Foot and Ankle Society (AOFAS) Ankle-Hindfoot score provided a direct *in vivo* clinical functional assessment. Improvements in pain, function, and mobility reflected the contribution of hAM augmentation to tendon repair outcomes. Thus, our findings can be regarded as complementary to prior preclinical investigations, extending the evaluation of hAM functionality into a clinical, patient-centered context.

This study has several limitations. First, its retrospective nature and non-randomized allocation of patients into the hAM and non-hAM groups may have introduced selection bias and limited the validity of direct comparisons between groups. Second, the sample size was relatively small. Third, due to incomplete medical records, the rupture sites of the Achilles tendon could not be determined in four patients (two in the hAM group and two in the non-hAM group). Fourth, the surgeries were performed by multiple surgeons, which may have introduced variability in surgical technique and outcomes. Fifth, while ultrasonography was used to confirm rupture, its operator-dependent nature and the inconsistent application of MRI, the gold standard for diagnosis, may have introduced diagnostic bias. Sixth, the absence of routine imaging, except in complicated cases, restricts the ability to fully evaluate tendon healing and represents a limitation of this study. Seventh, variability in surgeon experience and hAM application technique may affect operative duration. Lastly, due to the small sample size and the more recent clinical adoption of hAM products, there were significant differences between the two groups in terms of the laterality of the injured limb and the follow-up duration.

## 5 Conclusion

In this cohort study, the application of hAM allografts during Achilles tendon repair demonstrated comparable overall complication rates and functional outcomes to primary repair alone, while significantly reducing operative time. The findings also suggest that rupture sites located closer to the calcaneal insertion may be associated with an increased risk of postoperative infection, potentially due to anatomical and surgical factors. Although hAM products may provide certain intraoperative advantages, larger prospective studies with standardized protocols are needed to further assess their clinical efficacy and long-term outcomes in Achilles tendon repair.

## Data Availability

The original contributions presented in the study are included in the article/Supplementary Material, further inquiries can be directed to the corresponding author.
